# Study on the Secondary Recrystallization Process and Influencing Factors of 4N Pure Copper Wires

**DOI:** 10.3390/ma18235431

**Published:** 2025-12-02

**Authors:** Hao Xu, Xin Dong, Tianle Li, Zhixiang Qi, Guang Chen

**Affiliations:** 1 National Key Laboratory of High-end Equipment Casting Technology, Nanjing University of Science and Technology, Nanjing 210094, China; 2 Ningbo Branch of China Academy of Ordnance Science, Ningbo 315103, China

**Keywords:** pure copper wire, cold drawing, heat treatment, recrystallization, texture

## Abstract

**Highlights:**

**What are the main findings?**

- A cold drawing deformation over 89% is key to inducing secondary recrystallization.
- The grain coarsening temperature for 89% deformed wire is 400 °C, with rapid grain growth at 400–500 °C.
- Heat treatment temperature has a more significant impact on grain growth than holding time.

**What is the implication of the main finding?**

- A key processing window is identified for the new thermo-mechanical process.
- It provides theoretical support for enhancing the electrical conductivity of pure copper via thermomechanical processing.
- The dominant role of heat treatment temperature enables the mass production of highly conductive pure copper wires.

**Abstract:**

The transverse grain boundaries in pure copper wires increase resistivity, generating capacitance and inductance effects, leading to a decrease in the electrical conductivity of pure copper wires. Directional heat treatment technology can eliminate transverse grain boundaries in pure copper conductors, which is of great significance for improving electrical conductivity. Directional heat treatment is essentially a secondary recrystallization process, with influencing factors involving microstructure, texture, etc. This study systematically investigated the effects of cold-drawing deformation rate and heat treatment processes on secondary recrystallization microstructure, grain boundary structure, and crystallographic texture in pure copper wires. The results demonstrate that higher deformation levels (≥89%) facilitate secondary recrystallization and enhance <100> texture development. Moreover, the heat treatment temperature exerts a more significant influence on secondary recrystallization than the heat treatment duration. The grain coarsening temperature for pure copper wires with a deformation degree of 89% was determined to be 400 °C. These findings provide a fundamental basis for formulating directed heat treatment processes for pure copper wires.

## 1. Introduction

In the military field, information warfare is becoming increasingly important in modern warfare, with military electronic devices developing toward higher transmission efficiency. In the civilian sector, new energy vehicles have already become a national strategic industry, and motor performance is closely related to copper coils and copper wires. Improving the electrical conductivity of pure copper wires is an urgent issue to be addressed in the aforementioned fields.

Copper-based materials characterized by high electrical conductivity and superior strength have garnered significant attention in the energy and power sectors. Researchers such as Gao et al. have investigated in situ graphene-reinforced copper wires [1], while Wu et al. have explored the effects of cold deformation and heat treatment on the properties of Cu-Ag alloy wires [2]. However, the relatively high cost of raw materials for Cu-Ag alloys and the complex preparation processes associated with graphene/copper composites pose challenges for their widespread application. In contrast, pure copper conductors remain highly relevant in engineering applications and research due to their exceptional conductivity, well-established and scalable manufacturing processes, and excellent cost-effectiveness.

Currently, there are two methods to improve the electrical conductivity of pure copper wires: one is to increase purity, and the other is to reduce transverse grain boundaries. Researchers can use advanced technology to produce pure copper wires with a purity exceeding 5 N, but due to technical limitations, 8 N is the maximum.

Ingots produced by traditional continuous casting technology exhibit significant casting defects, such as pores, shrinkage cavities, and shrinkage porosity, may even lead to ingot deformation. The single crystal continuous casting technology demonstrates significant advantages. Gan et al. produced a CuNi10Fe1Mn alloy by the Ohno Continuous Casting (O.C.C.) technique, achieving a plasticity of 51%, a level unattainable with other casting technologies [3]. Yin et al. prepared CuAlNi alloy wires with 1.5 mm long columnar crystal structures using the O.C.C. technique [4]. Guo et al. successfully produced 4 mm-diameter single-crystal aluminum wires using the O.C.C. technique, achieving elongation and reduction in area values of 111.5% and 575%, respectively [5]. Zhang et al. produced 5 mm long columnar crystal Mg-Al eutectic alloy wires using the O.C.C. technique [6].

Using single-crystal continuous casting technology to eliminate transverse grain boundaries and produce single-crystal wires has become a new option [7,8]. However, single-crystal copper wires prepared in this way are generally larger than 1 mm, making them unsuitable for industrial applications [9,10].

Therefore, single-crystal copper wires obtained through continuous casting must undergo drawing before practical application, but this process leads to grain fragmentation and performance degradation [11,12,13]. Li et al. investigated the transformation of properties in single-crystal copper wires during cold deformation [14], discovering that the resistivity of copper wires generally exhibits an S-shaped increasing trend with greater deformation, with resistivity increasing by 4.9% after cold deformation. The research by Wang and Zhang et al. demonstrated an “S”-shaped relationship between wire resistivity and plastic deformation [15,16]. Wang et al. discovered that during the drawing of industrial single-crystal pure copper wires, the columnar grain orientation shifts from <100> to <111>, accompanied by a reduction in wire plasticity [17].

The earliest application of directional heat treatment technology was the preparation of single-crystal tungsten wires [18]. Later, it was gradually discovered that many metallic materials could obtain columnar or single-crystal structures through directional heat treatment [19,20,21,22]. Chou et al. found that for oxide dispersion strengthened superalloys such as MA956 and MA957, a columnar grain structure can be obtained under directional heat treatment conditions [23]. The existing research results indicate that cold-rolled polycrystalline nickel can form a columnar grain structure after directional heat treatment. Research by Li et al. has confirmed that the directional heat treatment of cold-rolled polycrystalline nickel is a secondary recrystallization process, during which most grain growth is hindered, and only a very few grains can grow rapidly [24]. Li et al. discovered that the columnar grains obtained from directionally heat-treated cold-rolled polycrystalline nickel exhibited a distinct {124} <211> orientation, and the boundaries between columnar grains tended to form low-energy small-angle grain boundaries or twin boundaries [25]. Greaves et al., in their study of directional heat treatment of nickel-based superalloys, found that the preferred orientation of the directionally grown columnar grains differed from that of the primary recrystallization texture [26]. Vallejos et al. designed a directional heat treatment apparatus and successfully obtained low-carbon steel with bamboo-joint-like directional microstructure using directional heat treatment technology [27].

Directional heat treatment technology is expected to become a new method for preparing pure copper wires with columnar grain or single crystal structures. Researchers have already used directional heat treatment to produce industrial pure iron with large aspect ratio columnar grains [28]. Employing directional heat treatment to eliminate transverse grain boundaries and prepare pure copper wires with large aspect ratio columnar grains or even single crystal structures holds significant importance. Our further research on pure copper conductors demonstrates that through appropriate directional heat treatment, the aspect ratio of columnar grains formed by secondary recrystallization can reach seven, accompanied by a 5% improvement in electrical conductivity [29,30], confirming the viability of this approach.

The key to achieving an ideal columnar grain structure lies in a profound understanding and effective control of the secondary recrystallization process. Secondary recrystallization behavior is influenced by various factors, primarily categorized into internal factors, such as the level of deformation and heat treatment temperature, and external factors, such as impurities, surface oxide film thickness, or intermetallic inclusions.

The deformation level is one of the key internal factors influencing secondary recrystallization. Prior to heat treatment, the material must undergo a drawing process to introduce a certain degree of pre-deformation, which provides the necessary driving force for secondary recrystallization. Yang et al. [31] investigated the effect of cold drawing on the microstructure of low-oxygen copper wire, finding that as the tensile strain increased, the grains were elongated and refined along the drawing direction, accompanied by dislocation multiplication and dynamic recrystallization, with grain boundaries tending to align parallel to the drawing direction. Research by Park et al. [32] indicated that a higher deformation level is more conducive to the occurrence of secondary recrystallization.

Heat treatment temperature is another critical internal factor. Highly deformed pure copper conductors exhibit a relatively lower recrystallization temperature. Wright et al. [33] observed a secondary recrystallization reaction occurring at a relatively low temperature (around 400 °C) in highly drawn wires (≥99.9%), which is distinct from several other types of secondary recrystallization that may occur within the range of 800 °C to 1000 °C.

Furthermore, external factors such as surface oxide film thickness, defects, or the content of intermetallic inclusions also exert a certain influence on secondary recrystallization. Jiang et al. [34] pointed out that impurity elements significantly inhibit grain boundary migration, leading to a reduction in recrystallized grain size. The study by Jakani et al. [35] demonstrated that an increase in sulfur impurity content prolongs the incubation period of recrystallization.

Given the dominant roles of deformation level and heat treatment temperature in the secondary recrystallization process, this study will systematically focus on these two core factors. To this end, we investigate the influence of cold drawing deformation and directional heat treatment processes on the microstructure of pure copper wires, explores the secondary recrystallization process of pure copper wires and its influencing factors, and analyzes the evolution patterns of microstructure, grain boundary structure, and crystallographic texture during isothermal annealing of pure copper wires.

## 2. Experiments

The experimental raw material was T1M20 copper billet with a purity of 99.99% and an original diameter of 8 mm. Copper wires with diameters of 1 mm, 0.85 mm, and 0.7 mm were obtained through drawing, with deformation amounts of 87%, 89%, and 91%, respectively.

Firstly, isothermal annealing experiments were conducted on pure copper conductors with different cold drawing deformation rates. Isothermal heat treatment was conducted using a commercially available KSL-1100X-J small box furnace(KJ Material Technology Co., Ltd., Hefei, China). The procedure involves first heating the box furnace to the set temperature, then placing the experimental material inside for a holding period, followed by its removal and air cooling.

**Figure 1:**
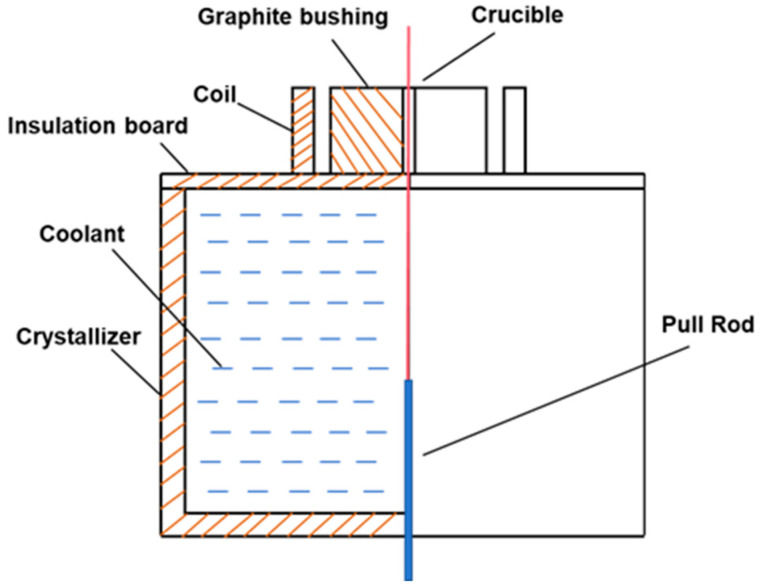
Schematic diagram of the internal structure of the high-frequency induction directional solidification furnace.

Secondly, directional heat treatment experiments were conducted under various conditions. These experiments employed a high-frequency induction directional solidification furnace (Zhengzhou Hengtong Furnace Industry Co., Ltd., Zhengzhou, China). The chamber of this system was connected to a mechanical pump and a molecular pump to achieve a high-vacuum environment. The heating system, centered around a high-frequency induction power supply, allowed for precise temperature control by adjusting the supply current. A custom-made graphite sleeve served as an indirect heat source, which was heated by a single-turn induction coil. With a wall thickness of 5 mm, this graphite sleeve effectively shielded against electromagnetic interference and, due to the small gap with the coil, provided high heating efficiency. Inside the furnace, a cooling crystallizer (Zhengzhou Hengtong Furnace Industry Co., Ltd., Zhengzhou, China) contained a Ga-In-Sn alloy. By adjusting the height of the coolant level and using an aluminum silicate plate for insulation between the coolant and the graphite sleeve, a significant temperature gradient was established at the front end of the hot zone. A withdrawal system, driven by a servo motor(Siemens AG, Munich, Germany), controlled the movement of the sample within a ceramic crucible through a pull rod at a controlled rate through the graphite hot zone; this ceramic crucible also acted to isolate and protect the pure copper wire from being wetted by the Ga-In-Sn alloy. Temperature measurement was achieved by inserting a type K thermocouple probe (Omega Engineering Inc., Norwalk, CT, USA) into the ceramic crucible(Yixing Borui Liante Precision Ceramics Co., Ltd., Yixing, China), which moved along with the withdrawal rod. A schematic diagram is shown in Figure 1.

To study the microstructure, the sample was first ground sequentially with 240#, 400#, 600#, 800#, and 1200# water sandpaper, then polished with 0.5 μm and 0.05 μm Al_2_O_3_ polishing solutions, followed by etching. The etchant was prepared with a ratio of 10 g FeCl_3_ + 100 mL HCl + 200 mL H_2_O, with an etching time of 3–5 s. The optical microscope used was the DM6M model from Leica, Germany, and the scanning electron microscope was the Auriga FIB/SEM dual-beam system from Zeiss, Germany. For the EBSD experiment, the sample tilt angle was 70°, the acceleration voltage was set at 20 kV, the working distance was 18 mm, and data processing was performed using version 4.4 of the Channel 5 software package developed by HKL Technology.

## 3. Results and Discussion

### 3.1. Effect of Cold Drawing Deformation on Secondary Recrystallization Microstructure of Pure Copper Wire

**Figure 2:**
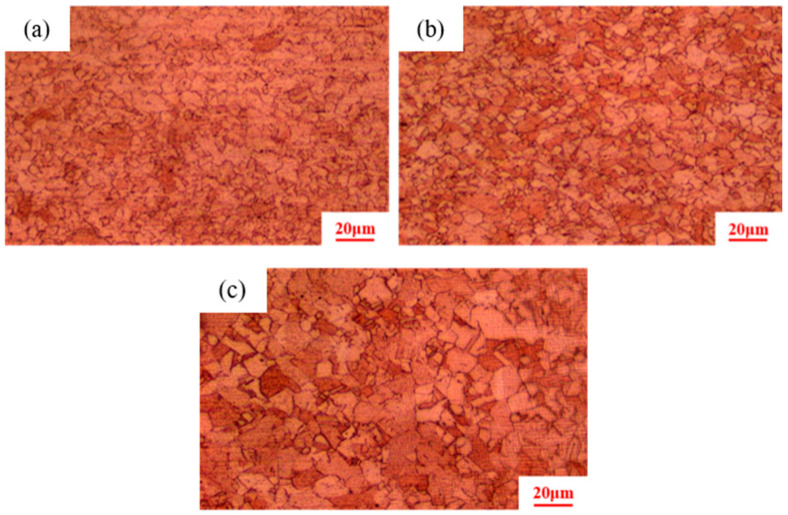
Initial microstructure of pure copper wires with different deformation amounts: (**a**) 91%; (**b**) 89%; (**c**) 87%.

Figure 2 shows that after isothermal annealing, the initial microstructures of pure copper conductors with different cold drawing deformation rates are all composed of fine equiaxed crystals. Statistical analysis reveals average grain sizes of 2 ± 0.15 μm, 3 ± 0.18 μm, and 5 ± 0.20 μm, respectively.

**Figure 3:**
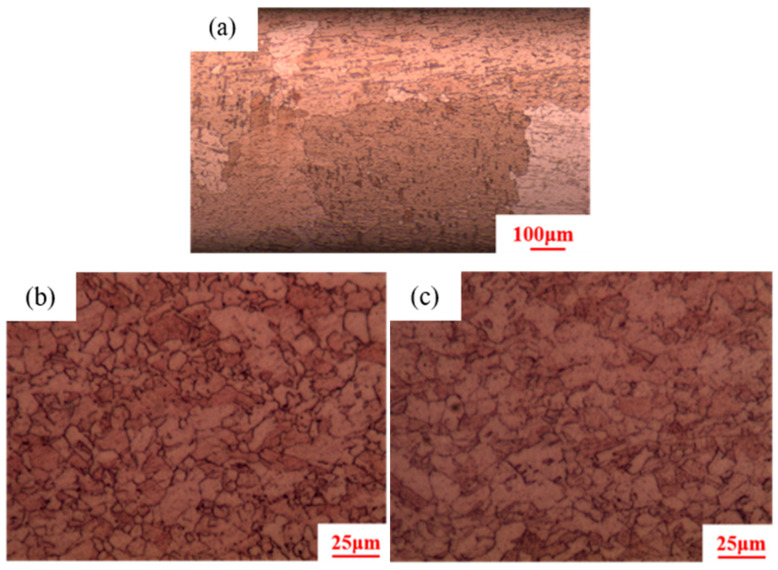
Microstructure of pure copper wires after heat treatment at 380 °C for 30 min with different deformation amounts: (**a**) 91%; (**b**) 89%; (**c**) 87%.

Using an isothermal heat treatment process of 380 °C × 30 min, it is found that the microstructure corresponding to 91% deformation consisted of coarse grains with an average diameter of 400 μm, while the microstructures corresponding to 89% and 87% deformation are still equiaxed grains, with average grain sizes of 5 ± 0.19 μm and 7 ± 0.23 μm, respectively, as shown in Figure 3. This indicates that during heat treatment at 380 °C, only the pure copper wire with 91% deformation underwent secondary recrystallization.

**Figure 4:**
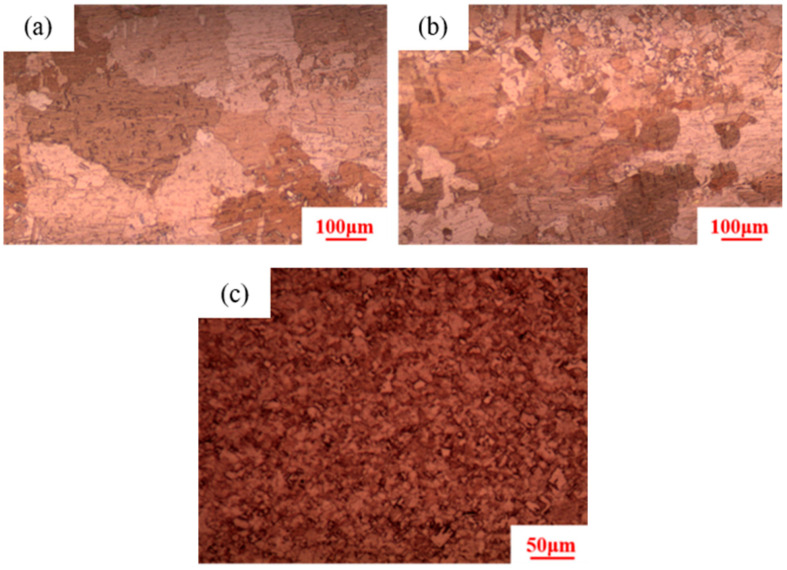
Microstructure of pure copper wire with different deformation amounts after heat treatment at 480 °C for 30 min: (**a**) 91%; (**b**) 89%; (**c**) 87%.

Figure 4 indicates that as the heat treatment temperature increases from 380 °C to 480 °C, secondary recrystallization also occurs in the pure copper wire with 89% deformation. The microstructure consists of large and small grains, with the large grains measuring 220 μm. The grain size of the pure copper wire with 91% deformation decreases to 200 μm. The microstructure of the pure copper wire with 87% deformation still consists of fine equiaxed grains.

**Figure 5:**
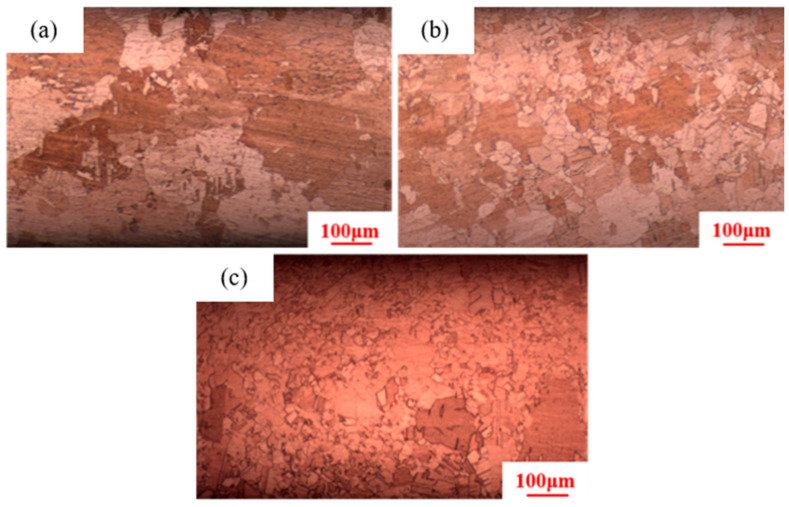
Microstructures of pure copper wires with different deformation amounts after annealing at 580 °C for 30 min: (**a**) 91%; (**b**) 89%; (**c**) 87%.

As the heat treatment temperature increases to 580 °C (Figure 5), secondary recrystallization occurs in all pure copper wires regardless of deformation degree. For wires with 87% deformation, the maximum grain size remains below 150 μm after 580 °C × 30 min heat treatment, while those with 91% and 89% deformation exhibit grain sizes of 180 μm and 200 μm, respectively.

The above results indicate that the deformation amount has a significant impact on the secondary recrystallization structure of pure copper wire. As the deformation decreases, the secondary recrystallization process slows down, the number of abnormally grown grains decreases, and the grain size becomes smaller. The fundamental reason is that during the deformation process of pure copper wires, grain rotation occurs, and the orientation tends to be consistent. The greater the degree of deformation, the more pronounced the preferred orientation, and the stronger the anisotropy of grain boundary migration during heat treatment. Taking a deformation of 91% as an example, the preferred orientation of grain boundary migration is the strongest, with a few grains growing rapidly, while most grains are suppressed. The grains with a fast growth rate have ample space to grow, forming large-sized grains. For a deformation of 87%, the preferential orientation of grain boundary migration is weakened, the temperature required for secondary recrystallization increases, the size of abnormally grown grains decreases, resulting in an inconspicuous secondary recrystallization phenomenon.

### 3.2. Effect of Heat Treatment Process on the Secondary Recrystallization Microstructure of Pure Copper Wires

#### 3.2.1. Effect of Annealing Temperature on Secondary Recrystallization Microstructure in Pure Copper Wires

The empirical formula for secondary recrystallization in pure metals is as follows:

T = (0.5 ~ 0.8) Tm(1)

**Figure 6:**
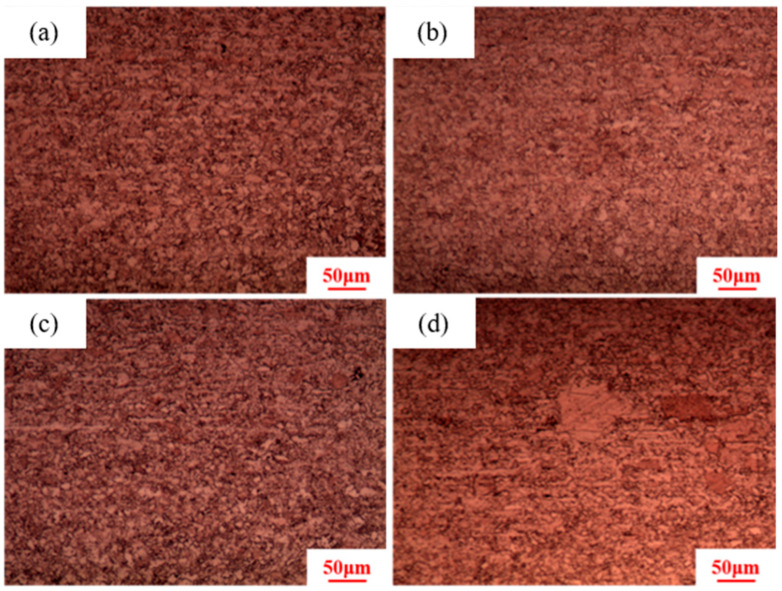
Microstructure of 89% deformed pure copper wire after heat treatment at different temperatures for 30 min: (**a**) 370 °C; (**b**) 380 °C; (**c**) 390 °C; (**d**) 400 °C.

Calculations indicate that the grain coarsening temperature of pure copper conductors is approximately 380–400 °C. Therefore, the holding temperatures selected are 370 °C, 380 °C, 390 °C, and 400 °C, with a holding time of 30 min and a deformation amount of 89%. As shown in Figure 6, the microstructures after heat treatment at 370 °C, 380 °C, and 390 °C are all fine equiaxed grains, while the microstructure after heat treatment at 400 °C contains large equiaxed grains surrounded by small grains. According to the criteria for abnormal grain growth [36,37], abnormal grain growth occurs at 400 °C. Statistical data shows that the large grain size is 100 μm, while the small grain size is 5 μm. Therefore, it can be concluded that the grain coarsening temperature for pure copper wire with 89% deformation is 400 °C.

**Figure 7:**
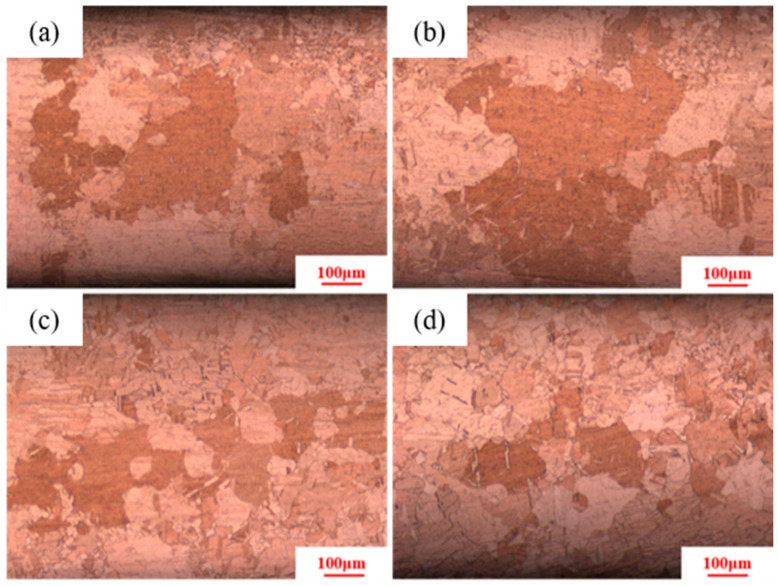
Microstructure of 89% deformed pure copper wire after heat treatment at different temperatures for 30 min: (**a**) 450 °C; (**b**) 500 °C; (**c**) 550 °C; (**d**) 600 °C.

**Figure 8:**
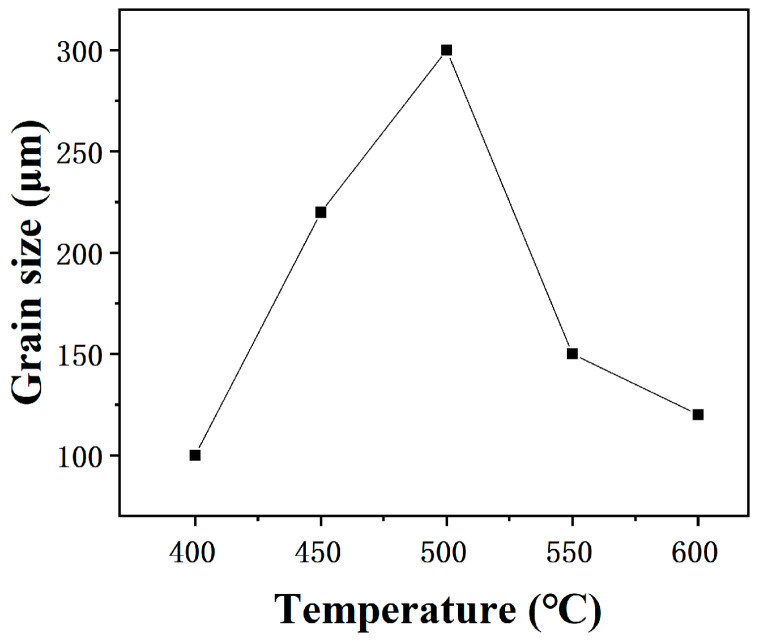
Maximum grain size of 89% deformed pure copper wire after heat treatment at different temperatures for 30 min.

Figure 7 shows the microstructure of the pure copper wire with 89% deformation after heat treatment at different temperatures for 30 min. The maximum grain size under different conditions can be statistically obtained from the graph. Continuing to increase the holding temperature, it is found that when the temperature reaches 450 °C, the maximum grain size reaches 220 μm; when the temperature reaches 500 °C, the maximum grain size reaches 300 μm; when the temperature reaches 550 °C and 600 °C, the maximum grain size decreases to 150 μm and 120 μm, as shown in Figure 8. When the heat treatment temperature is slightly higher than the grain coarsening temperature, a few grains undergo secondary recrystallization, and the tendency for grain growth increases. As the temperature rises, the grain boundary migration rate accelerates, and the grain size increases. At higher temperatures, the number of secondary recrystallized grains increases, competition intensifies, the tendency for grain growth decreases, and the grain size becomes smaller.

#### 3.2.2. Effect of Holding Time on Secondary Recrystallization Microstructure of Pure Copper Wire

**Figure 9:**
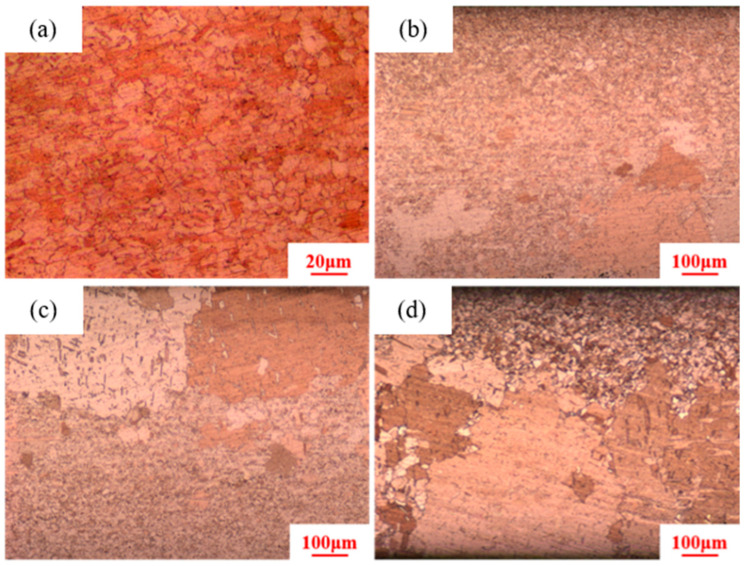
Microstructure of 89% deformed pure copper wire after heat treatment at 400 °C for different holding times: (**a**) 2 min; (**b**) 40 min; (**c**) 90 min; (**d**) 120 min.

To investigate the TYeffect of holding time on secondary recrystallization in pure copper wires, pure copper wires with 89% deformation are selected, with holding temperatures set at 400 °C, 550 °C, and 700 °C. When the holding temperature is 400 °C, the holding times are set to 20 min, 40 min, 90 min, and 120 min, as shown in Figure 9. The results show that after 20 min of heat treatment, the pure copper wire exhibits uniform grains with a size of 5 μm. According to previous research findings, grain growth only begins after 30 min. After 40 min of heat treatment, the maximum grain size reaches 330 μm. When the heat treatment time increases to 90 min and 120 min, the maximum grain size reaches 400 μm and 500 μm, respectively, with small equiaxed grains gradually decreasing.

**Figure 10:**
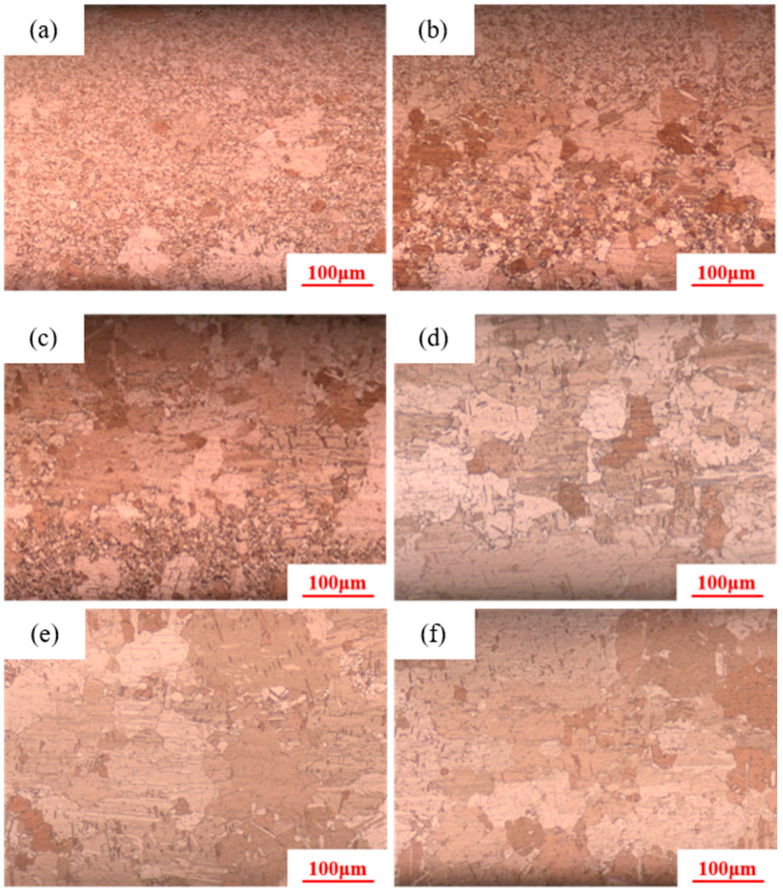
Microstructure of 89% deformed pure copper wire after heat treatment at 550 °C with different holding times: (**a**) 1 min; (**b**) 3 min; (**c**) 5 min; (**d**) 8 min; (**e**) 10 min; (**f**) 20 min.

When the holding temperature increases to 550 °C, the holding times are set to 1 min, 3 min, 5 min, 8 min, 10 min, and 20 min, as shown in Figure 10. When the holding time is 1 min, secondary recrystallization has already occurred in the pure copper wire, with a grain size of 100 μm. After heat treatments of 3 min, 5 min, and 8 min, the maximum grain sizes reach 120 μm, 150 μm, and 180 μm, respectively, while the number of small grains decreased. When the holding time reaches 10 min and 20 min, there are basically only large grains, and secondary recrystallization is essentially complete.

**Figure 11:**
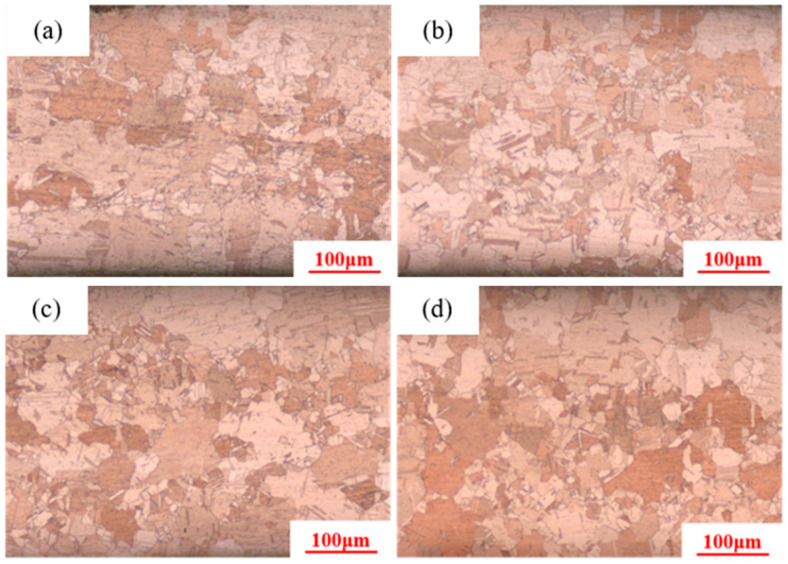
Microstructure of 89% deformation pure copper wire after heat treatment at 700 °C with different holding times: (**a**) 10 s; (**b**) 30 s; (**c**) 1 min; (**d**) 10 min.

Figure 11 shows the microstructure of pure copper wire after holding at 700 °C for 10 s, 30 s, 1 min, and 10 min. It can be seen that small grains basically disappear within 10 s, indicating that the pregnancy period for secondary recrystallization of pure copper wire at 700 °C is shorter. Even with increased holding time, the microstructure changes very little.

The above experimental results indicate that the pregnancy period for secondary recrystallization in pure copper wires varies with heat treatment temperature: 30 min at 400 °C, reduces to 1 min at 550 °C, and only a few seconds at 700 °C, demonstrating that the higher the annealing temperature, the shorter the pregnancy period for secondary recrystallization in pure copper wires.

### 3.3. Evolution of Grain Boundary Structure and Crystallographic Texture in Secondary Recrystallized Pure Copper Wire

#### 3.3.1. Initial Grain Boundary Structure and Crystallographic Texture of Pure Copper Wire

**Figure 12:**
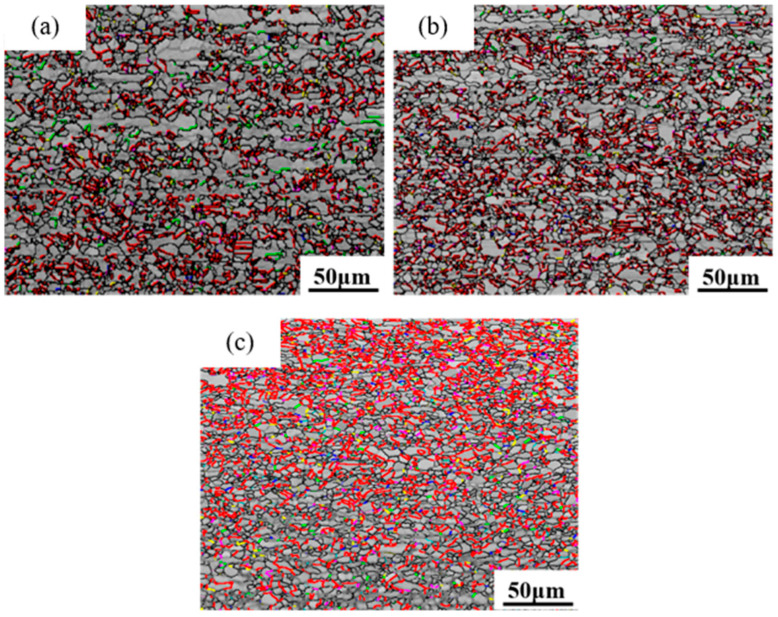
Reconstruction diagram of initial grain boundaries in pure copper wires with different deformation amounts: (**a**) 91%; (**b**) 89%; (**c**) 87%. Gray: low-angle grain boundaries; Black: high-angle grain boundaries; Red: Σ3 boundaries; Green: Σ5 boundaries; Blue: Σ7 boundaries; Pink: Σ9 boundaries; Yellow: Σ11 boundaries.

Grain boundaries are primarily classified into high-angle grain boundaries and low-angle grain boundaries, with high-angle grain boundaries further including general random high-angle grain boundaries and special grain boundaries [38]. According to the definition of grain boundaries, low-angle grain boundaries have misorientation angles of less than 15°, while high-angle grain boundaries have misorientation angles greater than 15°. In EBSD experiments, the minimum angle for grain boundary detection is set at 2°. As shown in Figure 12, the gray lines represent low-angle grain boundaries, the black lines represent high-angle grain boundaries, the red lines represent Σ3 grain boundaries, the green lines represent Σ5 grain boundaries, the blue lines represent Σ7 grain boundaries, the pink lines represent Σ9 grain boundaries, and the yellow lines represent Σ11 grain boundaries. The lower the Σ value, the greater the number of coincident lattice sites, the lower the grain boundary mobility, and the smaller the grain boundary energy.

**Figure 13:**
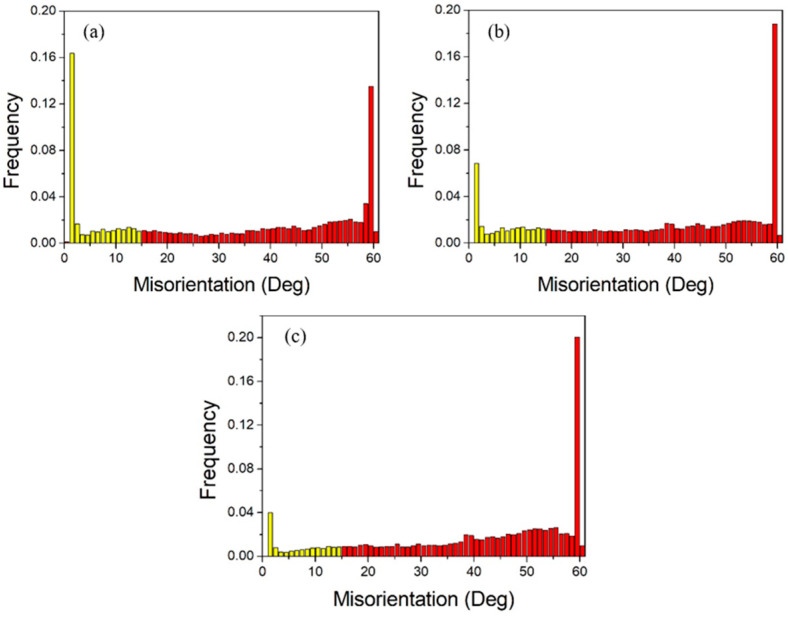
Statistical diagram of grain boundary angles in the initial microstructure of pure copper wires with varying deformation amounts: (**a**) 91%; (**b**) 89%; (**c**) 87%. Yellow: low-angle grain boundaries; Red: high-angle grain boundaries. Figure 14 presents statistics on special grain boundaries in pure copper wires with three different deformation levels. The results show that Σ3 grain boundaries are the most prevalent. In pure copper wires with 91% and 89% deformation, Σ5 grain boundaries rank second in frequency, while in wires with 87% deformation, Σ9 grain boundaries are the second most common.

Figure 13 shows the grain boundary angles of pure copper wires with three different deformation amounts. Yellow represents low-angle grain boundaries, and red represents high-angle grain boundaries. It can be observed that high-angle grain boundaries are predominant. The grain boundary angles exhibit a bimodal distribution, with the first peak near 2° and the second peak near 60°, indicating that low-angle grain boundaries of 2° and high-angle grain boundaries of 60° are the most prevalent. As the deformation increases, the first peak becomes more pronounced, while the second peak diminishes.

**Figure 14:**
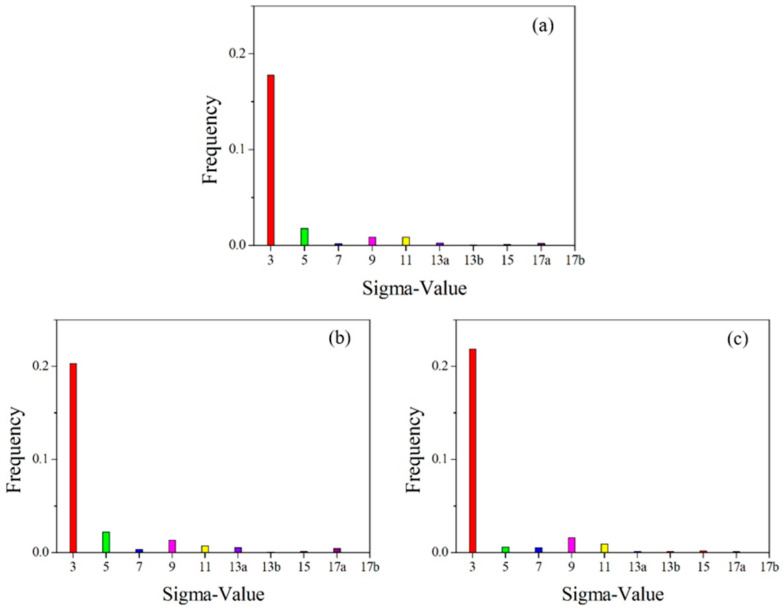
Statistical chart of special grain boundaries in the initial microstructure of pure copper wires with different deformation levels: (**a**) 91%; (**b**) 89%; (**c**) 87%. Red: Σ3 boundaries; Green: Σ5 boundaries; Blue: Σ7 boundaries; Pink: Σ9 boundaries; Yellow: Σ11 boundaries.

The greater the degree of deformation, the more small-angle grain boundaries and the fewer large-angle grain boundaries. According to statistical data, small-angle grain boundaries account for 30.9% at 91% deformation, and 22.3% and 13.1% at 89% and 87% deformation, respectively. With decreasing deformation, the proportion of low-angle grain boundaries (2°) decreases from 16.2% to 6.8% and 4.1%, while the proportion of high-angle grain boundaries (60°) increases from 13.6% to 18.5% and 20.2%.

**Figure 15:**
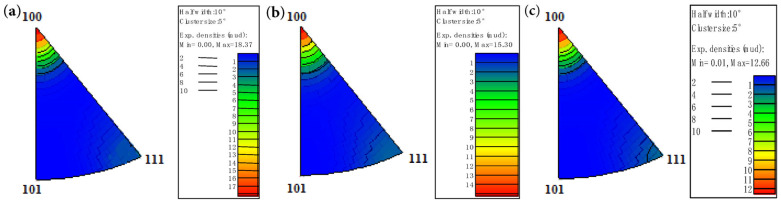
Inverse pole figures along the drawing direction of the initial microstructure of pure copper wires with different deformation amounts: (**a**) 91%; (**b**) 89%; (**c**) 87% [30].

Figure 15 shows that pure copper wires with different deformation levels all exhibit strong <100> texture. The 91% deformed pure copper wire demonstrates the strongest <100> texture, while the 87% deformed pure copper wire shows the weakest <100> texture, along with the appearance of <111> texture.

**Figure 16:**
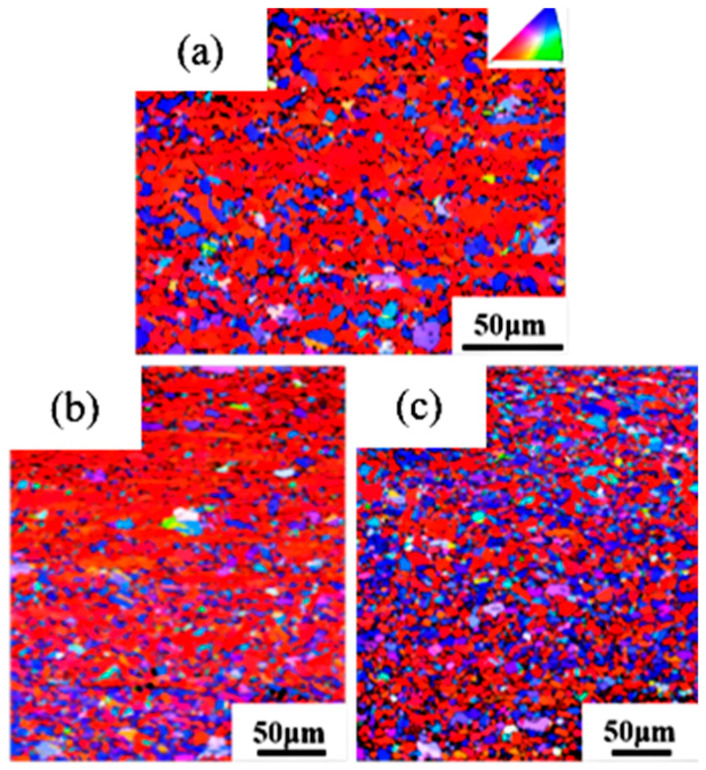
Orientation distribution diagram of the initial microstructure of pure copper wires with different deformation amounts along the drawing direction: (**a**) 91%; (**b**) 89%; (**c**) 87%.

In Figure 16, different colors represent different orientations: red indicates <100>, green represents <101>, and blue denotes <111>. The <100>-oriented grains are densely distributed in the 91% and 89% deformation pure copper wires, while the <100> and <111> oriented grains are more dispersed in the 87% deformation pure copper wire.

#### 3.3.2. Grain Boundary Structure and Crystallographic Texture of Secondary Recrystallization in Pure Copper Wires

**Figure 17:**
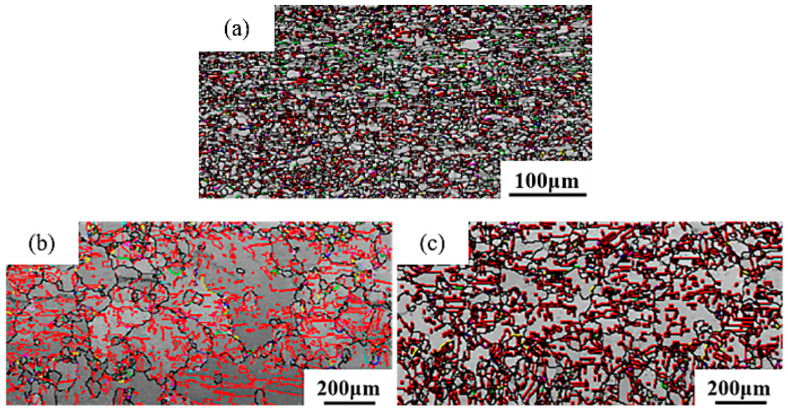
Grain boundary reconstruction diagrams of pure copper wire with 89% deformation after heat treatment at different temperatures for 30 min: (**a**) 400 °C; (**b**) 500 °C; (**c**) 600 °C. Gray: low-angle grain boundaries; Black: high-angle grain boundaries; Red: Σ3 boundaries; Green: Σ5 boundaries; Blue: Σ7 boundaries; Pink: Σ9 boundaries; Yellow: Σ11 boundaries.

Firstly, the effect of heating temperature on the grain boundary structure and crystallographic texture of secondary recrystallization is investigated. Figure 17 shows the grain boundary reconstruction diagram. Based on previous experimental results, it is found that secondary recrystallization begins after 89% deformed pure copper wire undergoes heat treatment at 400 °C for 30 min, is nearly completed after 500 °C for 30 min, and fully completed after 600 °C for 30 min.

**Figure 18:**
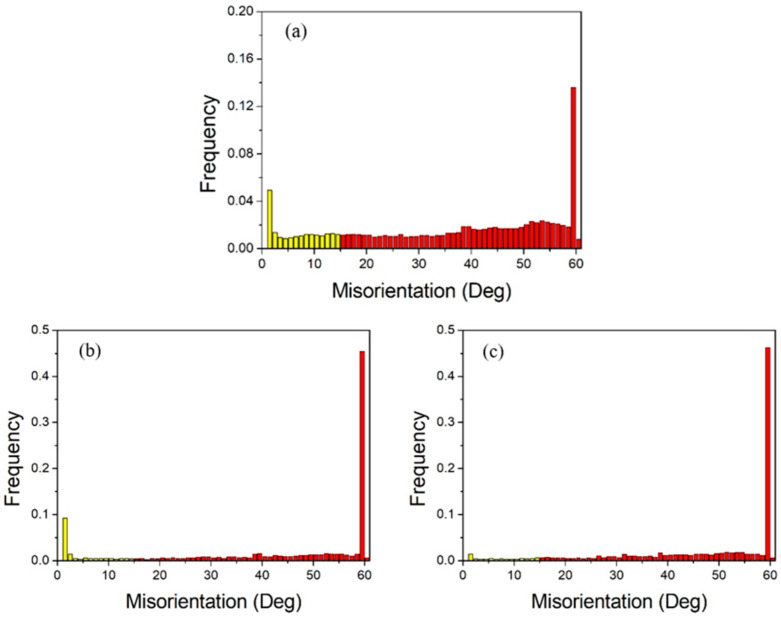
Statistical diagram of grain boundary angles for 89% deformed pure copper wire after heat treatment at different temperatures for 30 min: (**a**) 400 °C; (**b**) 500 °C; (**c**) 600 °C. Yellow: low-angle grain boundaries; Red: high-angle grain boundaries.

Figure 18 shows the grain boundary angle distribution statistics. At 400 °C, the grain coarsening temperature, most grains remain fine and equiaxed after 30 min of heat treatment, resulting in a higher proportion of low-angle grain boundaries accounting for 20.3% of total grain boundaries, with 60° grain boundaries comprising 13.8% of high-angle grain boundaries. After heat treatment at 500 °C, the proportion of low-angle grain boundaries decreases to 16.2% of total grain boundaries, while the proportion of 60° grain boundaries among high-angle grain boundaries increases to 46.1%. When the heat treatment temperature reaches 600 °C, the proportion of low-angle grain boundaries further decreases to 7.2%, while the proportion of 60° grain boundaries remains essentially unchanged.

**Figure 19:**
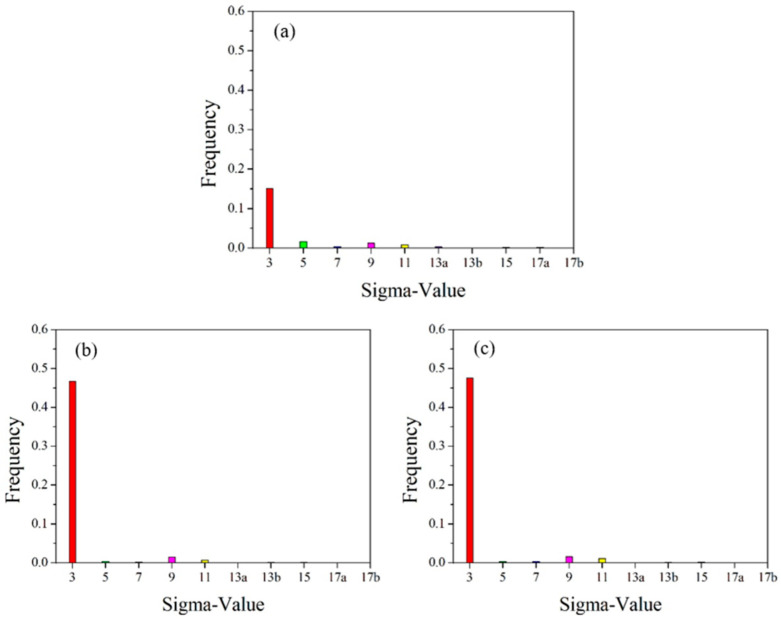
Statistical diagram of special grain boundaries for 89% deformed pure copper wire after heat treatment at different temperatures for 30 min: (**a**) 400 °C; (**b**) 500 °C; (**c**) 600 °C. Red: Σ3 boundaries; Green: Σ5 boundaries; Blue: Σ7 boundaries; Pink: Σ9 boundaries; Yellow: Σ11 boundaries.

Figure 19 shows the statistical distribution of special grain boundaries. The 400 °C heat-treated microstructure exhibits the highest proportion of Σ3 grain boundaries among special grain boundaries, followed by Σ5 grain boundaries, similar to the initial microstructure. After heat treatment at 500 °C, a significant number of Σ3 grain boundaries form, while Σ5 grain boundaries nearly disappear, with Σ9 grain boundaries becoming the second most prevalent special grain boundary. Following 600 °C heat treatment, the proportion of Σ3 grain boundaries remains nearly unchanged at 47.1%. These experimental results demonstrate that as heat treatment temperature increases, low-angle grain boundaries decrease, while high-angle grain boundaries increase. Since grain boundary migration is a thermally activated process, grain boundary mobility increases exponentially with rising temperature. As Σ3 and Σ9 grain boundaries possess relatively low grain boundary energy and are less prone to migration, the formation of high-angle grain boundaries becomes favorable for reducing free energy.

**Figure 20:**
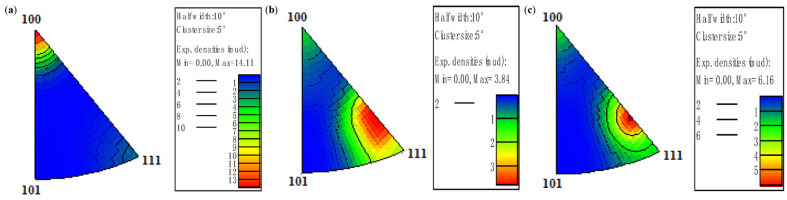
Inverse pole figures along the drawing direction of 89% deformed pure copper wire after heat treatment at different temperatures for 30 min: (**a**) 400 °C; (**b**) 500 °C; (**c**) 600 °C.

Figure 20 shows the inverse pole figure. The pure copper wire exhibits a strong <100> texture with an intensity level of 14.11 after 400 °C heat treatment. Following 500 °C heat treatment, the <100> texture weakens significantly, while a new <112> texture emerges with an intensity level of 3.84. After 600 °C heat treatment, the <112> texture intensity increases to 6.16. It can therefore be concluded that for pure copper wire with 89% deformation, the <100> texture gradually transforms into a <112> secondary recrystallization texture as the heat treatment temperature increases.

**Figure 21:**
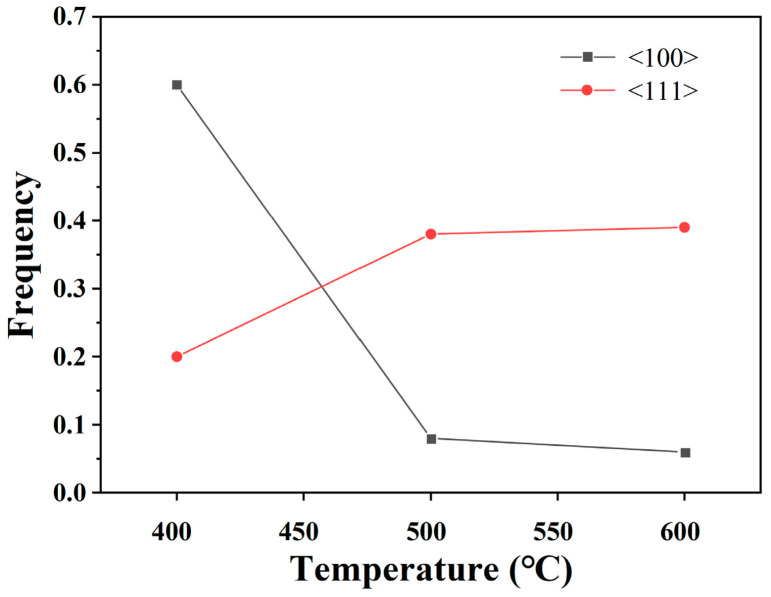
Variation in typical texture with heat treatment temperature for 89% deformed pure copper wire after 30 min heat treatment.

Figure 21 shows the quantitative statistics of typical textures. As the heat treatment temperature increases, the <100> texture weakens, while the <111> texture strengthens. From 400 °C to 500 °C, the texture changes significantly: the <100> texture decreases from 0.6 to 0.08, and the <111> texture increases from 0.2 to 0.38. From 500 °C to 600 °C, the textures stabilize, with the <100> texture at 0.06 and the <111> texture at 0.39.

**Figure 22:**
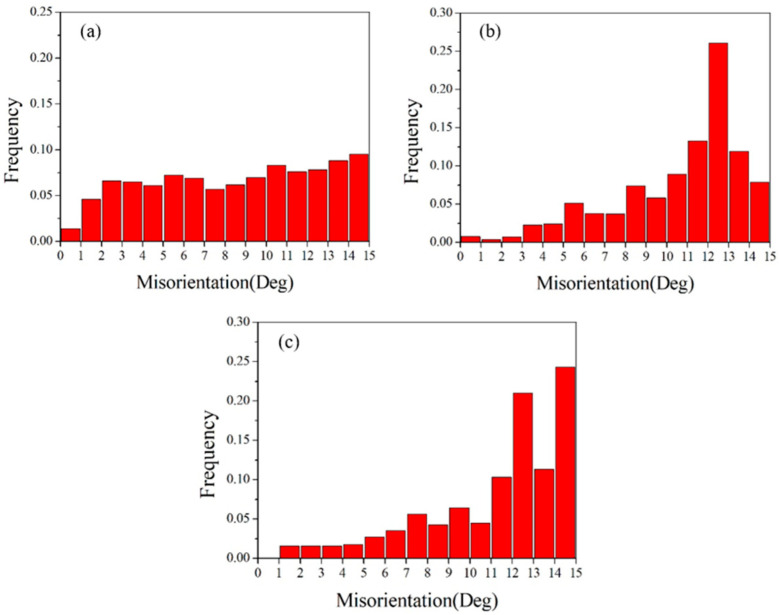
Deviation degree of <111> texture in 89% deformed pure copper wire after heat treatment at different temperatures for 30 min: (**a**) 400 °C; (**b**) 500 °C; (**c**) 600 °C.

Figure 22 shows the degree of deviation in <111> texture, where grains with deviation angles within 15° are considered textured. As temperature increases, the <111> texture intensifies, but the degree of deviation also increases. Combined with Figure 19, it can be observed that <112> texture gradually forms during the heating process.

**Figure 23:**
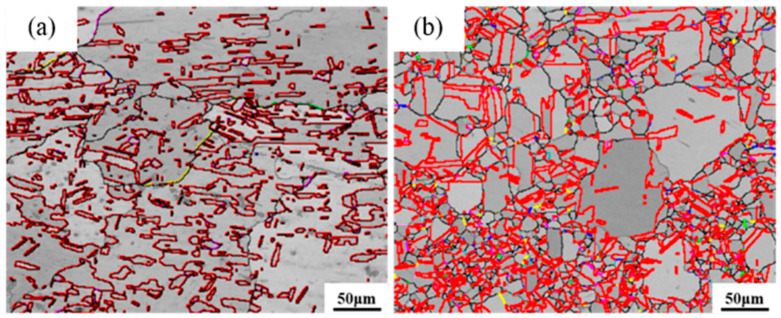
Grain boundary reconstruction diagrams of pure copper wires with different deformation amounts after heat treatment at 600 °C for 30 min: (**a**) 91%; (**b**) 87%.

Secondly, the influence of deformation on the grain boundary structure and crystallographic texture of secondary recrystallization is investigated. Figure 23 shows the reconstructed grain boundaries of pure copper wires after heat treatment at 600 °C for 30 min, where gray represents low-angle grain boundaries and red indicates twin boundaries. The pure copper wire with 91% deformation exhibits larger grain sizes after heat treatment, containing numerous low-angle grain boundaries and twin boundaries. The pure copper wire with 87% deformation shows relatively small grain sizes after heat treatment, containing abundant twin boundaries.

**Figure 24:**
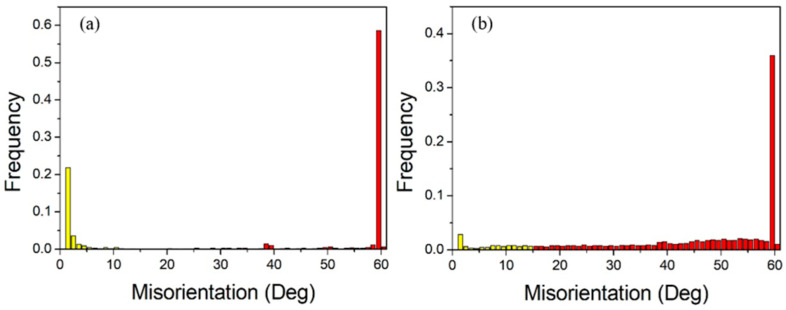
Statistical diagram of grain boundary angles for pure copper wires with different deformation amounts after heat treatment at 600 °C for 30 min: (**a**) 91%; (**b**) 87%. Yellow: low-angle grain boundaries; Red: high-angle grain boundaries.

**Figure 25:**
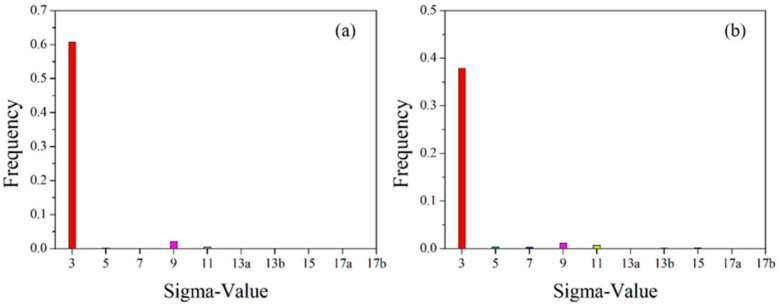
Statistical diagram of special grain boundaries in pure copper wires with different deformation amounts after heat treatment at 600 °C for 30 min: (**a**) 91%; (**b**) 87%. Red: Σ3 boundaries; Green: Σ5 boundaries; Blue: Σ7 boundaries; Pink: Σ9 boundaries; Yellow: Σ11 boundaries.

Figure 24 shows the grain boundary angle distribution, while Figure 25 presents the special grain boundary statistics. In heat-treated pure copper wire with 91% deformation, low-angle grain boundaries account for 31.1% of total grain boundaries, with twin boundaries constituting 60.3% of high-angle grain boundaries. For heat-treated pure copper wire with 87% deformation, low-angle grain boundaries represent less than 10% of total grain boundaries, while twin boundaries make up 37.5% of high-angle grain boundaries. The driving force for grain growth originates from the reduction in interfacial energy, whereby heat treatment generates numerous low-energy Σ3 boundaries that facilitate system energy minimization.

**Figure 26:**
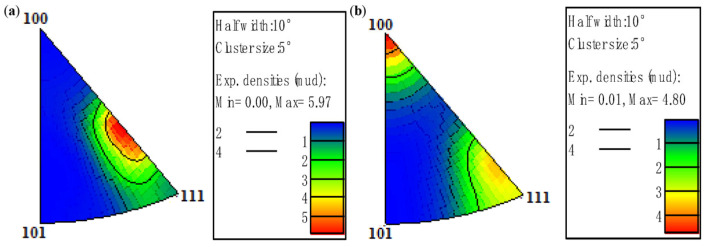
Inverse pole figures along the drawing direction of pure copper wires with different deformation amounts after 600 °C heat treatment for 30 min: (**a**) 91%; (**b**) 87% [30].

Zorina [39] also employed EBSD technology to discover a certain relationship between the recrystallized grain orientation of copper and its deformation texture. Figure 26 shows the inverse pole figure of pure copper wire after heat treatment at 600 °C for 30 min. The primary texture of 87% deformed pure copper wire is <100>, with weaker <111> texture. The 91% deformed pure copper wire exhibits stronger <112> texture, consistent with that of 89% deformed pure copper wire, indicating that secondary recrystallization in pure copper wires forms <112> texture.

## 4. Conclusions

This study systematically investigates the effects of cold drawing deformation and heat treatment processes on the secondary recrystallization microstructure, grain boundary structure, and crystallographic texture of pure copper wires, and finds that:

1. The greater the deformation of pure copper wire, the more likely secondary recrystallization is to occur. When the deformation is below 89%, secondary recrystallization is difficult to initiate.

2. Temperature has a significant effect on secondary recrystallization in pure copper wires. The grain coarsening temperature for pure copper wires with 89% deformation is 400 °C. When the holding temperature is between 400 °C and 500 °C, the grain size of secondary recrystallization increases rapidly with rising temperature. When the holding temperature exceeds 500 °C, the number of secondary recrystallized grains increases, while the grain size decreases with increasing temperature.

3. The effect of holding time on secondary recrystallization of pure copper conductors depends on temperature. At lower holding temperatures, the holding time has a more significant influence on secondary recrystallization, while this effect gradually diminishes as the holding temperature increases.

4. Pure copper wires with different cold drawing deformation amounts all exhibit a <100> texture along the drawing direction; the greater the deformation, the more pronounced the <100> texture becomes. During the secondary recrystallization process, the <100> texture of the pure copper wires gradually transforms into a <112> texture.

## Data Availability

The original contributions presented in this study are included in this article. Further inquiries can be directed to the corresponding authors.
